# Lysophosphatidic Acid Alters the Expression Profiles of Angiogenic Factors, Cytokines, and Chemokines in Mouse Liver Sinusoidal Endothelial Cells

**DOI:** 10.1371/journal.pone.0122060

**Published:** 2015-03-30

**Authors:** Chia-Hung Chou, Shou-Lun Lai, Cheng-Maw Ho, Wen-Hsi Lin, Chiung-Nien Chen, Po-Huang Lee, Fu-Chuo Peng, Sung-Hsin Kuo, Szu-Yuan Wu, Hong-Shiee Lai

**Affiliations:** 1 Department of Surgery, National Taiwan University Hospital and National Taiwan University College of Medicine, Taipei, Taiwan; 2 Graduate Institute of Toxicology, National Taiwan University College of Medicine, Taipei, Taiwan; 3 Department of Oncology, National Taiwan University Hospital and National Taiwan University College of Medicine, Taipei, Taiwan; 4 Department of Radiation-Oncology, Wan Fang Hospital, Taipei Medical University, Taipei, Taiwan; University of Bari Medical School, ITALY

## Abstract

**Background and Aims:**

Lysophosphatidic acid (LPA) is a multi-function glycerophospholipid. LPA affects the proliferation of hepatocytes and stellate cells in vitro, and in a partial hepatectomy induced liver regeneration model, the circulating LPA levels and LPA receptor (LPAR) expression levels in liver tissue are significantly changed. Liver sinusoidal endothelial cells (Lsecs) play an important role during liver regeneration. However, the effects of LPA on Lsecs are not well known. Thus, we investigated the effects of LPA on the expression profiles of angiogenic factors, cytokines, and chemokines in Lsecs.

**Methods:**

Mouse Lsecs were isolated using CD31-coated magnetic beads. The mRNA expression levels of LPAR’s and other target genes were determined by quantitative RT-PCR. The protein levels of angiogenesis factors, cytokines, and chemokines were determined using protein arrays and enzyme immunoassay (EIA). Critical LPAR related signal transduction was verified by using an appropriate chemical inhibitor.

**Results:**

LPAR1 and LPAR3 mRNA’s were expressed in mouse LPA-treated Lsecs. Treating Lsecs with a physiological level of LPA significantly enhanced the protein levels of angiogenesis related proteins (cyr61 and TIMP-1), cytokines (C5/C5a, M-CSF, and SDF-1), and chemokines (MCP-5, gp130, CCL28, and CXCL16). The LPAR1 and LPAR3 antagonist ki16425 significantly inhibited the LPA-enhanced expression of cyr61, TIMP-1, SDF-1, MCP-5, gp130, CCL28, and CXCL16, but not that of C5/C5a or M-CSF. LPA-induced C5/C5a and M-CSF expression may have been through an indirect regulation mechanism.

**Conclusion:**

LPA regulated the expression profiles of angiogenic factors, cytokines, and chemokines in Lsecs that was mediated via LPAR1 and LPAR3 signaling. Most of the factors that were enhanced by LPA have been found to play critical roles during liver regeneration. Thus, these results may prove useful for manipulating LPA effects on liver regeneration.

## Introduction

LPA is a potent signaling lipid molecule that is involved in numerous phenomena, such as cell migration, preventing cellular apoptosis, angiogenesis, and others. LPA modulates its biological functions through the activation of at least six G-protein-coupled receptors (LPA1-6) [1]. Liver regeneration is an important phenomenon after liver injury, and the reproducibility of a partial hepatectomy model has made it the preferred approach for studies on liver regeneration [2]. Many studies have demonstrated that exogenous factors, such as pharmaceutical agents like acetaminophen [3], chemicals like CCl_4_ [4], and endogenous factors, such as angiotensinogen, IL-6, and interferon gamma receptors, are critically involved in liver regeneration [5–7].

Ikeda et al. first demonstrated that LPA might affect the proliferation of hepatocytes and stellate cells in those liver diseases that disrupted platelet activation [8]. Recently, by using a partial hepatectomy mouse model, Simo et al. found that liver regeneration after partial hepatectomy was associated with significant changes in circulating LPA levels (LPA increased significantly at 72 hours post- partial hepatectomy to 6.30 ± 0.67 μM as compared to 3.58 ± 0.37 μM in sham-operated mice) and that hepatic mRNA levels of LPAR1, LPAR3, and LPAR6 were expressed in a time- and cell-dependent manner [9]. Additionally, their immunohistochemical staining results revealed that LPAR1 protein was expressed in non-parenchyma cells, and that LPAR3 and LPAR6 proteins were widely distributed in regenerating liver tissue [9]. The study by Simo et al. clearly demonstrated the phenomena of increased LPA levels and expression levels of LPAR’s during liver regeneration.

LPA receptors were originally defined as an endothelium differentiation gene (edg) subfamily of G-protein-coupled receptors[10]. Liver sinusoidal endothelial cells have been found to play important roles during liver regeneration [11]. In this study, we used endothelial cell-specific marker CD31-coated magnetic beads to isolate liver sinusoidal endothelial cells from mice [12], and confirmed their purity by determining CD45 positive rates [13]. These liver sinusoidal endothelial cells were then used to investigate the effects of treating these cells with physiological LPA levels with regard to their expression of angiogenesis factors, cytokines, and chemokines by using proteome profile arrays.

## Materials and Methods

### Isolation of mouse liver sinusoidal endothelial cells

Our animal use protocols were reviewed and approved by the Institutional Animal Care and Use Committee (IACUC) of National Taiwan University College of Medicine and College (IACUC Approval No: 20120247). For primary cultures, mouse liver sinusoidal endothelial cells were isolated from 20 normal 6–8 week-old male C57BL/6 mice per experiment. The yields of mouse liver sinusoidal endothelial cells were, on average, 5 x 10^6^ sinusoidal endothelial cells per 1 g of liver.

A mouse was first anesthetized with 5% isoflurane inhalation and after sacrifice by C0_2_ asphyxiation; the entire liver was carefully removed. Liver tissues were washed twice with 50 ml of Dulbecco's modified Eagle's medium (DMEM; Invitrogen Technologies), cut into small pieces, and then incubated with 2.5 ml of 0.1% type II collagenase in DMEM at 37°C for 1.5 hours. A cell suspension was passed through a 100-um mesh and washed twice with 5 ml of Hanks balanced salts solution that contained 10% fetal calf serum (Invitrogen). Isolated cells were incubated in 1 ml of cold M199 medium (Invitrogen) that contained 1μg/ml of rabbit, anti-mouse CD31 antibody (sc-1506-R; Santa Cruz Biotechnology) for 30 minutes with gentle agitation, followed by adding 50 μl of Dynabeads M280 sheep, anti-rabbit IgG (10 mg/ml), and then incubated for an additional 30 minutes.

Endothelial cells that bound to the magnetic beads were removed from unbound non-endothelial cells by magnetic isolation using an MPC-1 magnet (Dynal, Oslo, Norway). The cells bound on beads were re-suspended and cultured in endothelial cell growth medium (Cell Applications, San Diego, CA, USA).

### Flow Cytometry Analysis

The following antibodies/reagents were used for flow cytometry analyses: anti-mouse CD31 (sc-1506-R; Santa Cruz Biotechnology), anti-mouse CD45 (sc-25590; Santa Cruz Biotechnology), and normal rabbit IgG (sc-3888; Santa Cruz Biotechnology), used as a negative control. Stained cells were analyzed by flow cytometry (BD FACSCalibur).

### Preparation of conditioned medium (CM)

Cultures of liver sinusoidal endothelial cells (2 × 10^6^/10-mm dish) were rinsed twice with PBS and then cultured in 5 ml of serum-free M199 with 5 μM LPA or vehicle for 24 hrs. Conditioned medium from liver sinusoidal endothelial cells was collected and clarified by centrifugation (10,000 rpm for 5 min at 4°C) to remove cellular debris prior to protein array analysis.

### LPA and chemical inhibitor

LPA (Oleoyl-L-α-lysophosphatidic acid sodium salt, L-7260) and ki16425 (SML0971) were purchased from Sigma (St. Louis, MO, USA).

### Protein arrays

Mouse angiogenesis, cytokine, and chemokine antibody arrays (Proteome Profiler, R&D Systems; Ary015, Ary006, and Ary017, respectively) were used to analyze angiogenesis factor, cytokine, and chemokine expression profiles according to the manufacturer’s instructions. Briefly, conditioned medium was first mixed with a biotinylated detection antibody cocktail at room temperature for 1 hour while the array membrane was blocked with blocking solution provided by the manufacturer. A digital imaging system (Bio Pioneer Tech Co., Ltd.) was used to detect chemiluminescent signals, which were further analyzed using ImageJ software.

### Enzyme Immunoassay (EIA)

Conditioned medium from liver sinusoidal endothelial cells was used for determinations of Cyr61, TIMP-1, C5/C5a, M-CSF, MCP-5, SDF-1, gp130, CCL28, and CXCL16 protein levels. These were determined using specific EIA kits (R&D Systems), according to the manufacturer’s instructions. Each measurement was performed in duplicate.

### Q-RT-PCR

Q-RT-PCR was performed as in a previous study (13). Briefly, total RNA was isolated from liver sinusoidal endothelial cells using RNAzol B reagent (Biotecx Laboratories, Houston, TX). Then, cDNA was prepared from 2 μg of total RNA using random hexamer primers (ImProm-II RT System; Promega, Southampton, UK). LPAR1-6, Cyr61, TIMP-1, C5/C5a, M-CSF, MCP-5, SDF-1, gp130, CCL28, and CXCL16 mRNA levels were determined with a quantitative real-time PCR detection system (Light Cycler DNA Master SYBR Green I; Roche Molecular Biochemicals, Indianapolis, IN). The primers used are shown in Table 1. The amplification program included an initial incubation at 61°C for 20 min, followed by 40 cycles of denaturation at 95°C for 10 s, annealing at 55–57°C for 10 s, and extension at 72°C for 10 s. The expression level of each mRNA was normalized to that of GAPDH mRNA.

**Table 1:** The primers for Q-RT-PCR.

Gene	Forward primer (5’~3’)	Reverse primer (5’~3’)
LPAR1	CTGCCTCTACTTCCAGCCCTGTAA	TGCTCACTGTGTTCCATTCTGTGG
LPAR2	GACCACACTCAGCCTAGTCAAGAC	CTTACAGTCCAGGCCATCCA
LPAR3	CCACTTTCCCTTCTACTACCTGCT	GACGGTCAACGTTTTCGACACC
LPAR4	CAGTGCCTCCCTGTTTGTCTTC	GAGAGGGCCAGGTTGGTGAT
LPAR5	TCCACGCTGGCTGTATATGG	TCGCGGTCCTGAATACTGTTC
LPAR6	GATCACTCTCTGCATCGCTGTTTC	CCCTGAACTTCAGAGAACCTGGAG
GAPDH	GCCATCAACGACCCCTTCAT	ATGATGACCCGTTTGGCTCC
Cyr61	AGACCCTGTGAATATAACTCCAGAA	AATTGCGATTAACTCATTGTTTCTC
TIMP-1	CAGCAAAGAGCTTTCTCAAAGACCT	TAGATAAACAGGGAAACACTGTGCA
C5/C5a	TACCAATGCCAACCTGGTGAAAGG	TCTGCAGAACCTCTTTGCCCATGA
M-CSF	TTGGCTTGGGATGATTCTCAG	GCCCTGGGTCTGTCAGTCTC
MCP-5	CCTGTGGCCTTGGGCCTCAA	GAGGTGCTGATGTACCAGTTGG
SDF-1	GAGAGCCACATCGCCAGAG	TTTCGGGTCAATGCACACTTG
gp130	TCATCAACAGAACCACGTCC	CCATACATGAAGTGCCATGC
CCL28	CAGGGCTCACACTCATGGCT	GCCATGGGAAGTATGGCCTTC
CXCL16	AAACATTTGCCTCAAGCCAGT	GTTTCTCATTTGCCTCAGCCT

### Statistical analysis

Results are given as means ± SD’s. Two-tailed t-tests were used to compare the results between the indicated groups in Results. A p-value of < 0.05 was considered significant.

## Results

### LPAR1, LPAR3, and LPAR6 are expressed in liver sinusoidal endothelial cells

**Fig 1:**
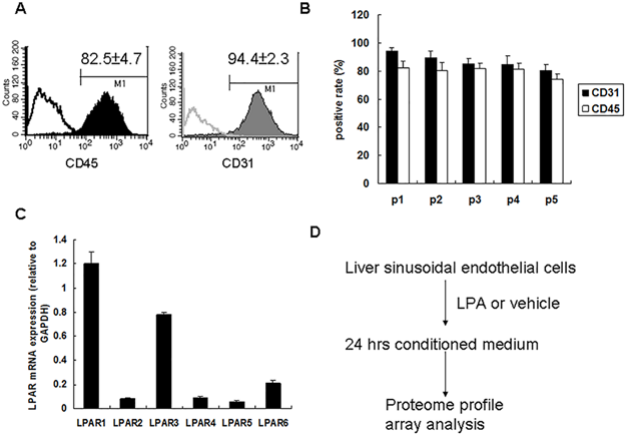
Liver sinusoidal endothelial cell isolation and LPA receptor expression patterns. (A) Liver sinusoidal endothelial cells were isolated from mouse liver tissue and their purity was determined by CD45 (left panel) and CD31 (right panel) positives rate by flow cytometry analysis. (B) Liver sinusoidal endothelial cell purity during five serial passages. (C) LPA receptors’ mRNA expression determined by qRT-PCR. LPAR1 and LPAR6 mRNA expression was normalized to that of *GAPDH* mRNA expression (n = 3). (D) Strategy for investigating LPA effects on liver sinusoidal endothelial cells using proteome profile arrays.

Because lysophosphatidic acid receptor (LPAR) expression has been shown to be involved during liver regeneration, we focused on the effects of LPA on liver sinusoidal endothelial cells. We isolated liver sinusoidal endothelial cells by using Dynabeads to positively isolate CD31-positive endothelial cells from mouse liver tissue. The purity of isolated liver sinusoidal endothelial cells was verified by determining the CD31 positive rate by flow cytometry and by expression of the hematopoietic cell marker CD45 that has also been reported to be expressed on liver sinusoidal endothelial cells (Fig 1A). The purities of liver sinusoidal endothelial cells during five serial passages were determined, which showed that from the first to the fifth passage, the CD31 positive rates were from 94.4 ± 2.3% to 80.3 ± 4% and the CD45 positive rates were from 82.5 ± 4.7% to 74.3 ± 3.9% (Fig 1B). For this study, liver sinusoidal endothelial cells were only used after the fifth passage.

We also determined the mRNA expressions of LPAR’s by qRT-PCR. This showed that LPAR1 and LPAR3 mRNA’s were strongly expressed and that LPAR6 mRNA was weakly expressed in liver sinusoidal endothelial cells (Fig 1C).

To determine the effects of LPA on liver sinusoidal endothelial cells, we screened the conditioned medium derived from LPA-treated liver sinusoidal endothelial cells for specific biological functions based on the results of proteome profile arrays (Fig 1D).

### LPA treatment enhances angiogenesis related factors Cyr61 and TIMP-1 expression in liver sinusoidal endothelial cells

**Fig 2:**
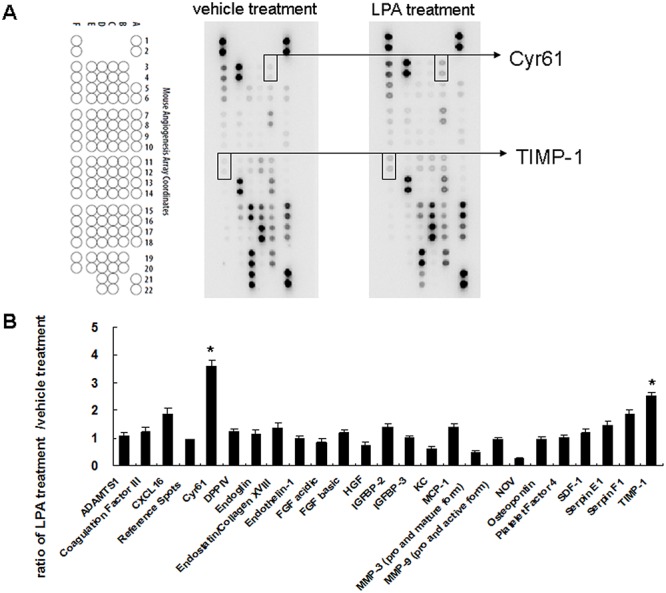
LPA effects on liver sinusoidal endothelial cells angiogenesis related protein expression. (A) Conditioned media derived from vehicle (1% BSA) and LPA (5 uM) treated liver sinusoidal endothelial cells were applied to angiogenesis protein array analysis. Data shown are representative images of three independent experiments. Significantly changed protein spots are indicated. (B) Quantitative results for angiogenesis protein arrays. Results are the ratios of LPA treatment versus vehicle treatment (n = 3). *, ratio of > 2 was defined as a significant change.

Conditioned media derived from liver sinusoidal endothelial cells after vehicle treatment or after LPA treatment were used to determine angiogenesis-related factors’ expression. When using an angiogenesis related protein array (Fig 2A), the spots for Cyr61 and TIMP-1 had different intensities between conditioned media after vehicle treatment and after LPA treatment. Quantitative results showed that Cyr61 and TIMP-1 expressions were 3.61 ± 0.2-fold and 2.53 ± 0.13-fold higher, respectively, with LPA treatment as compared to those with vehicle treatment (Fig 2B).

### LPA treatment enhances cytokine C5/C5a, M-CSF, MCP-5, and SDF-1 expression in liver sinusoidal endothelial cells

**Fig 3:**
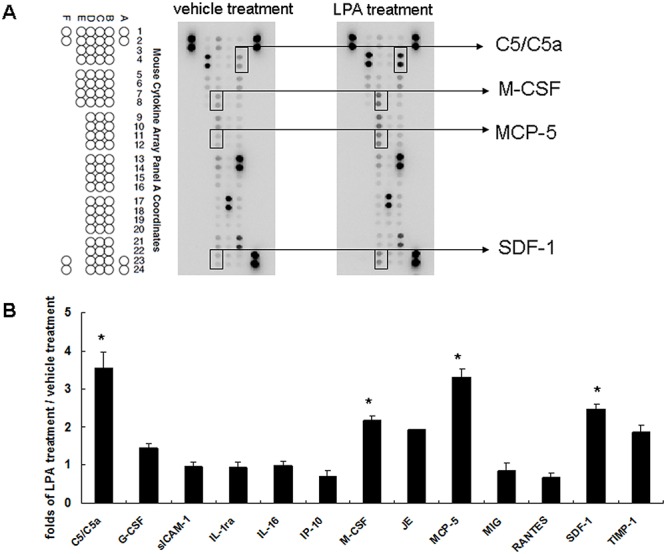
LPA effects on liver sinusoidal endothelial cells cytokine expression. (A) Conditioned media derived from vehicle (1% BSA) and LPA (5 uM) treated liver sinusoidal endothelial cells were applied to cytokine protein array analysis. Data shown are representative images of three independent experiments. Significantly changed protein spots are indicated. (B) Quantitative results for cytokine protein arrays. Results are the ratios of LPA treatment versus vehicle treatment (n = 3). *, ratio of > 2 was defined as a significant change.

Conditioned media derived from vehicle treated and LPA treated liver sinusoidal endothelial cells were used to determine cytokines’ expression using a cytokine protein array (Fig 3A). This showed that the spots for C5/C5a, M-CSF, MCP-5, and SDF-1 had different intensities between conditioned media after vehicle treatment and after LPA treatment. Quantitative results showed that LPA treatment had enhanced C5/C5a (3.56 ± 0.0.4-fold higher), M-CSF (2.17 ± 0.14-fold higher), MCP-5 (3.32 ± 0.21-fold higher), and SDF-1 (2.48 ± 0.13-foldhigher) expression relative to those after vehicle treatment (Fig 3B).

### LPA treatment enhances chemokine MCP-5, gp130, CCL28, and CXCL16 expression in liver sinusoidal endothelial cells

**Fig 4:**
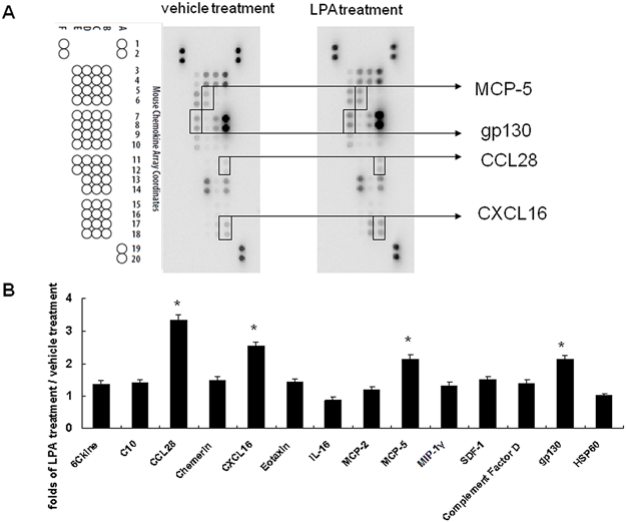
LPA effects on liver sinusoidal endothelial cells chemokine expression. (A) Conditioned media derived from vehicle (1% BSA) and LPA (5 uM) treated liver sinusoidal endothelial cells were applied to chemokine protein array analysis. Data shown are representative images of three independent experiments. Significantly changed protein spots are indicated. (B) Quantitative results for chemokine protein arrays. Results are the ratios of LPA treatment versus vehicle treatment (n = 3). *, ratio of > 2 was defined as a significant change.

Different conditioned media were also used to determine chemokines’ expression using a chemokine protein array (Fig 4A). This showed that the spots for MCP-5, gp130, CCL28, and CXCL16 had different intensities between conditioned media after vehicle treatment and after LPA treatment. Quantitative results showed that LPA enhanced MCP-5 (2.13 ± 0.13-fold higher), gp130 (2.12 ± 0.13-fold higher), CCL28 (3.33 ± 0.16-fold higher), and CXCL16 (2.53 ± 0.12-fold higher) expression relative to those after vehicle treatment (Fig 4B).

### LPA induced angiogenesis factor, cytokine, and chemokine expression in liver sinusoidal endothelial cells is mediated primarily through LPAR1 and LPAR3 signaling

**Fig 5:**
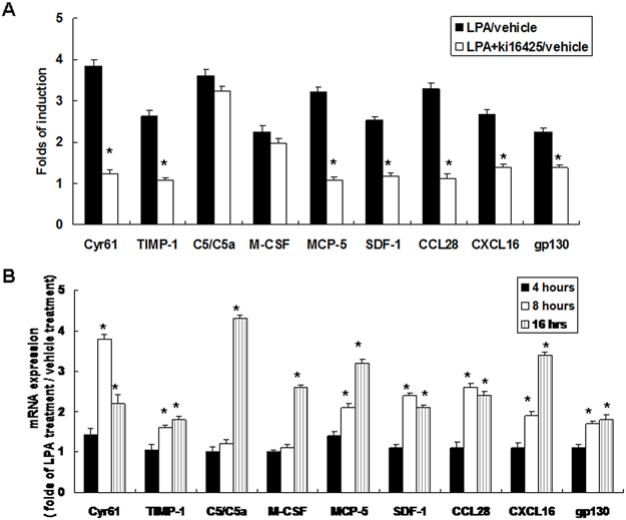
LPA effects on angiogenesis factor, cytokine, and chemokines expression are mediated by LPAR1 and LPAR3 signaling. (A) LPAR1 and LPAR3 signaling effects on LPA induced proteins level. Liver sinusoidal endothelial cells were pre-treated with an inhibitor of LPAR1 and LPAR3, ki16425 (1 uM), for 30 minutes prior 5 uM LPA treatment. After 24 hours, conditioned media derived from vehicle (1% BSA), LPA (5uM) alone, and ki16425 plus LPA treatment were collected for protein level determinations by EIA. Data shown are fold changes of induction with LPA alone versus vehicle treatment, and ki16425 treatment combined with LPA versus vehicle treatment. Results were compared between LPA treatment alone and ki16425 treatment combined with LPA (n = 3); *p < 0.05. (B) Time course for LPA effects on specific genes’ mRNA expressions. Liver sinusoidal endothelial cells were treated with vehicle (1% BSA) or LPA (5 uM). After 4, 8, and 16 hours, total RNA was isolated from vehicle and LPA treated cells for mRNA determinations by qRT-PCR. Data are fold changes of induction with LPA treatment versus vehicle treatment. Results were compared with vehicle treatment (n = 3); *p < 0.05.

LPA regulates several proteins based on the LPAR subtype. Our results (Fig 1) showed that LPAR1 and LPAR3 mRNA’s were strongly expressed in liver sinusoidal endothelial cells after LPA treatment. Thus, we investigated LPAR1 and LPAR3 involvement in LPA-mediated angiogenesis factor, cytokine, and chemokine expression in liver sinusoidal endothelial cells by using the specific chemical inhibitor ki16425. This showed that pre-treating cells with ki16425 significantly inhibited LPA enhanced expressions of angiogenesis factors (Cyr61 and TIMP-1), cytokines (MCP-5 and SDF-1), and chemokines (MCP-5, gp130, CCL28, and CXCL16). However, LPA enhanced cytokine C5/C5a and M-CSF expressions were not inhibited by ki16425 pre-treatment (Fig 5A).

To determine if LPA had a direct or an indirect effect on the expressions of these LPA induced angiogenesis factors, cytokines, and chemokines, the time course of LPA effects on these genes’ mRNA expressions were determined. This showed that the mRNA expressions for angiogenesis factors (Cyr61 and TIMP-1), cytokines (MCP-5 and SDF-1), and chemokines (MCP-5, gp130, CCL28, and CXCL16) were significantly increased after 8 hours of LPA treatment. By comparison, LPA enhanced cytokine C5/C5a and M-CSF mRNA expressions were significantly increased after 16 hours of LPA treatment (Fig 5B). Our results that ki16425 did not inhibit LPA enhanced C5/C5a and M-CSF protein expression and the late transcriptional regulation (16 hours) for LPA enhanced C5/C5a and M-CSF mRNA expression suggested that LPA might regulate C5/C5a and M-CSF through an indirect effect.

**Fig 6:**
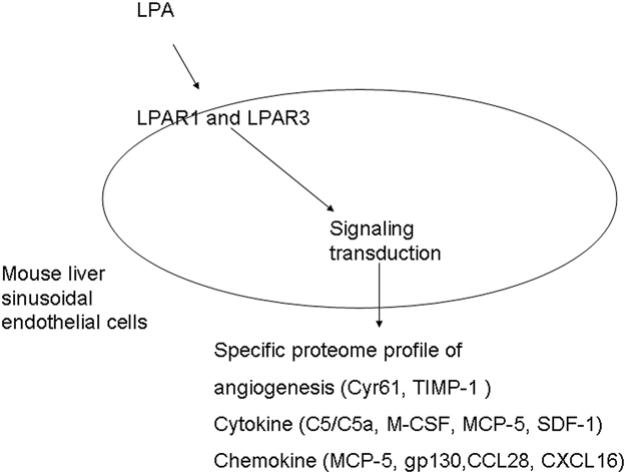
Schematic for LPA enhanced expression of angiogenesis factors, cytokines, and chemokines in liver sinusoidal endothelial cells mediated by LPAR1 and LPAR3 signaling.

Taken together, our results suggested that LPA might enhance several important angiogenesis factors, cytokines, and chemokines, including Cyr61, TIMP-1, C5/C5a, M-CSF, MCP-5, SDF-1, gp130, CCL28, and CXCL16, expression in liver sinusoidal endothelial cells that was mediated by LPAR1 and LPAR3 signaling (Fig 6).

## Discussion

Liver regeneration is an important phenomenon that reflects the reparative capacity of this vital organ. Several growth factors and cytokines, such as interleukin-6 [14, 15], tumor necrosis factor-α [16, 17], and hepatocyte growth factor [18, 19], have been found to be critically involved in liver regeneration, particularly in parenchymal cells. However, vascular endothelial growth factor (VEGF) and its receptors, flt-1 and KDR/flk-1, are expressed by non-parenchymal cells, including sinusoidal endothelial cells, in the liver after partial resection [20]. Using a sFlt-1-expressing adenoviral vector to infect C57BL6 mice to express the dominant negative receptor for VEGF and after 70% partial hepatectomy, a liver regeneration model showed that this angiogenesis inhibitor significantly suppressed hepatic regeneration [21]. By using VEGFR1 tyrosine kinase knockout mice, Ohkubo H et al. found that VEGFR1-expressing macrophages were recruited to the liver during hepatic ischemia/reperfusion and contribute to liver repair and sinusoidal reconstruction through regulating expression of pro-angiogenic factors. This study demonstrated that VEGFR1 activation is a potential therapeutic strategy for promoting liver repair and sinusoidal restoration after acute liver injury [22]. Coulon S et al. demonstrated that the blockage of VEGFR2 could attenuate steatosis and inflammation in a diet-induced mouse model for nonalcoholic steatohepatitis. The role of angiogenesis in the pathophysiology in nonalcoholic steatohepatitis may be worthwhile for a preventive and therapeutic setting [23]. By using an Innovative in vivo μCT methodology, Ehling J et al. found that CCL2-dependent infiltrating macrophages promote angiogenesis in progressive experimental liver fibrosis [24].

Liver sinusoidal endothelial cells are known to contribute to liver regeneration after liver injury [25]. In endothelial cell membranes, LPA is a well-known pleiotropic lipid molecule that has potent effects on cell migration [26] and membrane permeability [27]. The receptors for LPA that were first identified were designated the endothelium differentiation gene (edg) subfamily of G-protein-coupled receptors [28]. LPA has been found to primarily act through the activation of at least six G-protein-coupled receptors (LPA1-6) [29]. In this study, we found that LPAR1 and LPAR3 mRNA’s were strongly expressed and that LPAR6 mRNA was weakly expressed in mouse liver sinusoidal endothelial cells. Based on these findings for LPA receptors, we used a physiological level of LPA (5 uM) to stimulate liver sinusoidal endothelial cells for 24 hours. The conditioned media that were derived from these cell cultures were used for angiogenesis factor, cytokine, and chemokine expression profile determinations. Our results showed that LPA treatment enhanced Cyr61, TIMP-1, C5/C5a, M-CSF, MCP-5, SDF-1, gp130, CCL28, and CXCL16 expression in liver sinusoidal endothelial cells.

Cyr61 has been found to promote liver fibrosis regression through the induction of cellular senescence in hepatic myofibroblasts [30]. TIMP-1 knockout mice had impaired liver function and histological preservation after hepatic ischemia and reperfusion injury. Further, TIMP-1 expression promotes the survival and proliferation of liver cells, regulates leukocyte recruitment, and reduces active caspase-3 levels and increases Bcl-2 expression and Akt phosphorylation [31].

In C5-deficient mice, severely defective liver regeneration and persistent parenchymal necrosis were found after exposure to carbon tetrachloride. Additionally, murine C5 or C5a reconstitution in C5-deficient mice significantly restored hepatocyte regeneration after toxic injury, which results showed that C5/C5a contributed essentially to the early priming stages of hepatocyte regeneration [32, 33].

For osteopetrotic mice that genetically lack functional M-CSF, after these mice underwent 70% partial hepatectomy, the proliferation of hepatocytes was significantly impaired. However, when osteopetrotic mice were intraperitoneally administered mouse recombinant M-CSF before partial hepatectomy, the numbers of Kupffer cells were increased and liver regeneration was recovered [34].

In massive liver injury models, it was found that oval cell repair was involved in up-regulating the expression of SDF-1 in hepatocytes. The major biological role of SDF-1 is as a potent chemoattractant for hematopoietic cells homing to the liver after hepatic resection [35, 36, 37].

The interleukin (IL)-6 family of cytokines signal exclusively via the gp130 co-receptor, which subsequently dimerizes and initiates intracellular signaling [38]. Activation of IL-6/gp130-mediated STAT3 signaling pathway is crucial for both acute phase genes’ regulation after partial hepatectomy [39] and hepatic differentiation of adult bone marrow-derived mesenchymal stem cells [40].

In this study, we also investigated the possible signaling pathway involved in LPA enhanced Cyr61, TIMP-1, C5/C5a, M-CSF, MCP-5, SDF-1, gp130, CCL28, and CXCL16 expression in liver sinusoidal endothelial cells. Based on our findings for LPAR1 and LPAR3 mRNA expression, we used ki16425 that selectively inhibits LPAR1 and LPAR3 mediated actions [41]. These results showed that LPA enhanced C5/C5a and M-CSF expressions were not inhibited by ki16425. Combined with our mRNA level determination, we concluded that the regulation of LPA enhanced C5/C5a and M-CSF expression in liver sinusoidal endothelial cells may have been through LPAR1 and LPAR3 indirect regulation. Growth factors or cytokine could be regulated by autocrine effect, one factor may be first induced by LPA, following it may stimulate another factor to express through autocrine effect. Such as Lin CH et.al demonstrated that LPA-stimulated lymphangiogenesis in HUVECs is mediated through IL-1β-induced VEGF-C expression [42].

The results of this study clarified the expression of LPA receptors in mouse liver sinusoidal endothelial cells and showed that important angiogenesis factors, cytokines, and chemokines were regulated by LPA in mouse liver sinusoidal endothelial cells.
